# The Evolving Role of Genomic Testing in Early Breast Cancer: Implications for Diagnosis, Prognosis, and Therapy

**DOI:** 10.3390/ijms25115717

**Published:** 2024-05-24

**Authors:** Konstantinos Venetis, Carlo Pescia, Giulia Cursano, Chiara Frascarelli, Eltjona Mane, Elisa De Camilli, Elisabetta Munzone, Silvia Dellapasqua, Carmen Criscitiello, Giuseppe Curigliano, Elena Guerini Rocco, Nicola Fusco

**Affiliations:** 1 Division of Pathology, IEO, European Institute of Oncology IRCCS, 20141 Milan, Italy; konstantinos.venetis@ieo.it (K.V.); carlo.pescia@ieo.it (C.P.); giulia.cursano@ieo.it (G.C.); chiara.frascarelli@ieo.it (C.F.); eltjona.mane@ieo.it (E.M.); elisa.decamilli@ieo.it (E.D.C.); elena.guerinirocco@ieo.it (E.G.R.); 2 School of Pathology, University of Milan, 20122 Milan, Italy; 3 Department of Oncology and Hemato-Oncology, University of Milan, 20122 Milan, Italy; carmen.criscitiello@ieo.it (C.C.); giuseppe.curigliano@ieo.it (G.C.); 4 Division of Medical Senology, IEO, European Institute of Oncology IRCCS, 20141 Milan, Italy; elisabetta.munzone@ieo.it (E.M.); silvia.dellapasqua@ieo.it (S.D.); 5 Division of New Drugs and Early Drug Development for Innovative Therapies, IEO, European Institute of Oncology IRCCS, 20141 Milan, Italy

**Keywords:** early breast cancer, genomic testing, risk assessment, prognostication, adjuvant chemotherapy, endocrine therapy

## Abstract

Multigene prognostic genomic assays have become indispensable in managing early breast cancer (EBC), offering crucial information for risk stratification and guiding adjuvant treatment strategies in conjunction with traditional clinicopathological parameters. The American Society of Clinical Oncology (ASCO) guidelines endorse these assays, though some clinical contexts still lack definitive recommendations. The dynamic landscape of EBC management demands further refinement and optimization of genomic assays to streamline their incorporation into clinical practice. The breast cancer community is poised at the brink of transformative advances in enhancing the clinical utility of genomic assays, aiming to significantly improve the precision and effectiveness of both diagnosis and treatment for women with EBC. This article methodically examines the testing methodologies, clinical validity and utility, costs, diagnostic frameworks, and methodologies of the established genomic tests, including the Oncotype Dx Breast Recurrence Score^®^, MammaPrint, Prosigna^®^, EndoPredict^®^, and Breast Cancer Index (BCI). Among these tests, Prosigna and EndoPredict^®^ have at present been validated only on a prognostic level, while Oncotype Dx, MammaPrint, and BCI hold both a prognostic and predictive role. Oncologists and pathologists engaged in the management of EBC will find in this review a thorough comparison of available genomic assays, as well as strategies to optimize the utilization of the information derived from them.

## 1. Introduction

Multigene genomic assays are RNA-based molecular tests that assist treatment decision-making in early breast cancer (EBC) coupled with clinicopathological parameters [1,2,3]. Although assessing the standard variables (e.g., age, menopausal status, tumor size, lymph node status, histological grade, lymph vascular invasion, hormone receptor (HR), HER2, and Ki67 labeling index) can be highly informative for many patients, ~15% of women with stage I–III hormone receptor-positive (HR+) disease will go through metastatic events [4]. In patients with EBC, precisely estimating the possibility of local or distant recurrence is of vital importance for assessing the risk–benefit degree of systemic therapies [5,6]. In particular, to guide the selection and de-escalation of adjuvant therapy, there is a need for diagnostic algorithms that enhance the information obtained from histological criteria by integrating them with molecular data [7,8].

**Figure 1:**
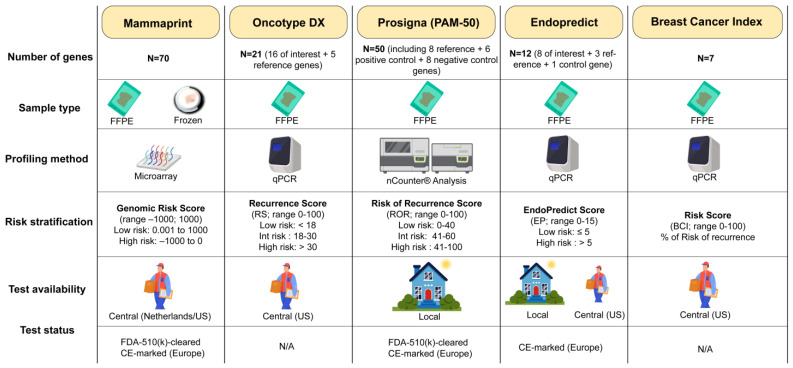
Overview of the RNA-based genomic assays currently used in early breast cancer clinical practice. FFPE, formalin-fixed paraffin-embedded; qPCR, quantitative polymerase chain reaction; BCI, Breast Cancer Index.

In the management of patients with EBC, several genomic tests are part of the comprehensive armamentarium of the multidisciplinary team [9,10]. These include Oncotype Dx, MammaPrint, Prosigna, EndoPredict^®^, and Breast Cancer Index (BCI), as summarized in Figure 1. Some of these assays not only offer prognostic information but also predict the potential benefit of additional chemotherapy, thus guiding the selection of adjuvant treatments [11]. Each assay evaluates unique sets of genes, employs distinct methodologies, and generates risk scores based on various parameters [12]. Furthermore, the execution of these tests can either be outsourced or performed in-house. Importantly, the clinical trials in which the aforementioned assays were validated differed significantly in terms of cohort compositions, including variations in patients’ menopausal and nodal status, HR expression, and endocrine therapies’ administration [12]. Therefore, the clinical validity and utility of these assays—whether prognostic, predictive of treatment benefit, or both—and the specific clinical contexts in which they are applicable (e.g., in patients with node-negative (N0) versus node-positive (N1) disease, or pre- versus postmenopausal individuals) vary significantly.

The most recent American Society of Clinical Oncology (ASCO) guidelines recommend the use of each genomic assay according to the level of evidence available for specific clinical conditions [11]. However, in certain N1 cases—such as EBC with four or more positive nodes and premenopausal patients with one to three positive nodes—a strongly recommended test is yet to be established. This gap highlights the need for a more integrated approach to genomic assays in routine clinical practice to address these unmet clinical needs. The present work aims to (i) provide a comprehensive overview and comparison of the clinically validated genomic assays in EBC, (ii) evaluate the current supporting evidence across different patient populations, and (iii) assess the impact of these assays on healthcare costs and treatment decision-making. By systematizing this information, the goal is to enhance understanding of the clinical utility and effectiveness of genomic assays, thereby facilitating personalized treatment strategies for individuals with EBC.

## 2. Testing Methodologies

### 2.1. Oncotype Dx^®^

Oncotype Dx (Exact Sciences Corp., Madison, WI, USA) is a first-generation 21-gene prognostic and predictive assay that has been prospectively validated for pre- and postmenopausal patients with N0 and N1 (1–3 metastatic lymph nodes) HR+/HER2− EBC [13]. Oncotype DX is a centralized test that involves the use of RT-qPCR to quantitatively assess the expression levels of 16 breast cancer-related genes, normalized to 5 reference genes [14]. The assay yields results in the form of a recurrence score (RS), which ranges from 0 to 100. The RS elucidates the probability of distant recurrence over a nine-year period (independently of clinicopathological characteristics) and prognosticates the potential benefits of adjuvant chemotherapy [15]. Cut-off points for RS were defined to categorize patients into groups with low (RS < 18), intermediate (18 ≤ RS ≤ 30), and high risk (RS > 30) [13,16]. In addition, high RS results (RS > 25) are predictive for adjuvant chemotherapy benefit [12]. The RSClin tool (accessible to healthcare professionals via https://online.genomichealth.com, accessed on 20 March 2024) integrates the RS with tumor grade, size, and patient age [17]. Developed based on data from a meta-analysis of 10,004 women with HR+/HER2-, N0 EBC, this tool provides more individualized information than clinical–pathological or genomic data alone [18].

### 2.2. MammaPrint^®^

MammaPrint (Agendia NV, Amsterdam, The Netherlands) is a first-generation 70-gene signature assay able to assess the risk of recurrence in EBC [19]. The test was designed using data from EBC patients who underwent surgery without receiving adjuvant systemic therapy, with a follow-up of 20 years [20]. MammaPrint is conducted in a centralized laboratory evaluating breast tumor tissue RNA expression, on both frozen and formalin-fixed paraffin-embedded (FFPE) samples, through microarray technology [21]. The results of the combined expression of 70 genes provide an index score between −1 and +1 and categorize tumors as low (index of 0.001 to 1) or high (index −1 to 0) risk of recurrence [22]. MammaPrint was the first assay to be cleared at the 510(k) level by the US FDA’s new in vitro diagnostic multivariate index assay classification and has the CE-IVD mark [23].

### 2.3. PAM50 Prosigna^®^

Prosigna (Veracyte, San Francisco, CA, USA) is a second-generation multigene expression assay, designed for assessing distant relapse-free survival (DRFS) a decade after breast cancer surgery [24]. The evaluation is carried out on the nCounter^®^ analysis system (NanoString Technologies, Inc., Seattle, WA, USA) using RNA extracted from FFPE breast tumor tissue samples [25]. Prosigna analyses a gene signature (PAM50) composed of 50 genes in order to classify surgically resected BC into four intrinsic subtypes (Luminal A, Luminal B, HER2-enriched, and basal-like). The PAM50 signature also includes eight housekeeping genes for normalization, along with six positive control genes and eight negative control genes [15,24]. The integrations of PAM50 gene expression profiling with proliferative information, and tumor size offer a risk-of-recurrence (ROR) score [24,26]. The ROR score, reported on a 0–100 scale, correlates with distant recurrence likelihood over 10 years for postmenopausal women with HR+, EBC not receiving chemotherapy [27]. In node-negative patients, the 10-year risk of recurrence is categorized as low (scores 0–40, with a risk of <5%), intermediate (scores 41–60, with a risk of ~10%), and high (scores 61–100, with a risk of >15%). For node-positive patients, the ROR is low (scores 0–40, with a risk of ~5%) and high (scores 41–100, with a risk of ~25%) [26]. Regulatory clearances permit test performance locally or in a centralized laboratory. The test holds FDA 510(k) clearance in the US and the Conformité Européene (CE) mark in Europe, suitable for FFPE tissue applications [25].

### 2.4. EndoPredict^®^

EndoPredict^®^ (Myriad Genetics, Salt Lake City, UT, USA) is a second-generation RNA-based 12-gene expression test that serves a prognostic and predictive function by determining the risk of distant recurrence up to 15 years and the 10-year absolute chemotherapy benefit for patients with ER+/HER2- primary breast cancer [28]. It can be conducted on FFPE tumor tissue (biopsies or surgical specimens) through RT-qPCR [29,30]. EndoPredict^®^ measures the activity of eight genes (proliferation-associated genes and HR-related genes for accurate assessment of early and late recurrence risk), three reference genes, and one control gene [31]. The resulting EP score, ranging from 0 to 15, categorizes patients with a score below or equal to 5 as low risk for distant recurrence under endocrine therapy and those with a score above 5 as high risk. The final EndoPredict^®^ result (EPclin Risk Score) (scale 1–6) is calculated by combining the 12-gene molecular score with the clinical risk factors of tumor size and lymph node status [28,30]. Higher EPclin scores indicate a higher risk of distant recurrence [29,30].

### 2.5. Breast Cancer Index^®^

The BCI (Biotheranostics Inc., San Diego, CA, USA) is a first-generation RT-PCR assay that relies on the analysis of seven genes to provide a prognostic risk of late distant recurrence (the result is given as a percentage) in addition to a yes/no prediction of benefit from receiving extended endocrine therapy in HR+ EBC [32]. Five cell cycle-associated genes that contribute to the molecular grade index (MGI) yield a quantitative and objective molecular assessment of tumor proliferative status [33]. The remaining two genes (HOXB13/IL17BR) provide a ratio value (H/I index ratio) that is associated with tumor responsiveness to endocrine therapy [34]. The BCI test is performed in a centralized laboratory on FFPE breast tumor tissue.

## 3. Clinical Validity and Clinical Utility

### 3.1. Oncotype Dx^®^

The landmark TAILORx trial (NCT00310180) played a significant role in elucidating Oncotype Dx significance. In this trial, patients with HR+/HER2- BC and an Oncotype-based RS of less than 11 exhibited favorable survival outcomes in the T1-2, N0 stage; endocrine therapy alone was also non-inferior to chemoendocrine therapy in the group of node-negative patients with RS ranging from 11 to 25 [35], while both menopausal and premenopausal patients with RSs ≥ 26 showed survival benefit from taxane and/or anthracycline-containing adjuvant chemotherapy [36]. The phase III West German Study Group (WSG) PlanB trial (NCT01049425) and the RxPONDER trial (NCT01272037) also tested the performance of Oncotype Dx RS on node-positive HR+/HER2− EBC, with similar results. The PlanB trial provided compelling evidence that in low-risk patients characterized by an Oncotype Dx RS of 11 or less and pN0-1 status, the omission of adjuvant chemotherapy resulted in excellent five-year disease-free survival (DFS) rates exceeding 94% and overall survival rates exceeding 99%. Notably, higher Ki-67, a marker of cellular proliferation, exerted an unfavorable impact on DFS, specifically in the RS > 25 subgroup, while its influence on prognosis was not observed in the RS ≤ 11 and RS 12–25 subgroups [37]. The RxPONDER trial further enriched our understanding of the utility of Oncotype Dx by prospectively randomizing HR+/HER2- EBC patients with 1–3 positive axillary lymph nodes and an RS of 25 or less to either endocrine therapy alone or chemoendocrine therapy. Among node-positive postmenopausal women with RS values of 25 or less, endocrine therapy alone proved sufficient, aligning with the findings from the PlanB trial. In contrast, node-positive premenopausal patients with RS values of 25 or less demonstrated improved outcomes with chemoendocrine therapy, showing longer invasive DFS and DRFS compared to endocrine therapy alone. This divergence in outcomes emphasized the importance of considering menopausal status when interpreting Oncotype Dx results in the node-positive HR+/HER2- EBC cohort [38]. Whether the chemotherapy benefit in premenopausal women was due to direct cytotoxic effects or chemotherapy-induced menopause remains unclear. To address this issue, the ongoing NRG-BR009 trial (NCT05879926) is currently ongoing. This phase III study aims to determine whether adjuvant chemotherapy (ACT) added to ovarian function suppression (OFS) plus endocrine therapy (ET) is superior to OFS plus ET in improving invasive breast cancer-free survival (IBCFS) among premenopausal patients with EBC HR+/HER2− and RS between 16 and 25 (for pN0) and between 0 and 25 (for pN1). Thus, Oncotype Dx is widely employed for tailoring treatment strategies in HR+/HER2− EBC, significantly influencing decisions based on genomic risk stratification.

### 3.2. MammaPrint^®^

The robust performance of MammaPrint has been substantiated through multiple studies, firmly establishing its prognostic and predictive capabilities. Notably, it excels at identifying patients with a low risk of recurrence, offering them the potential benefit of avoiding unnecessary chemotherapy [39,40]. The prognostic validity of MammaPrint received noteworthy validation through the RASTER study, marking the first prospective examination of its performance in patients with cT1-3N0M0 BC. This study rigorously evaluated the five-year distant-recurrence-free-interval (DRFI) probabilities, further attesting to the assay’s reliability and clinical relevance [41]. Furthermore, the multicenter randomized phase III MINDACT trial (NCT00433589) played a pivotal role in elucidating the clinical utility of MammaPrint as a guide for adjuvant chemotherapy decisions in EBC patients (pT1-3, pN0-1, pM0). Specifically, the study focused on patients with high clinical risk, as determined by the Adjuvant! Online tool, but low genomic risk based on the MammaPrint signature. Among these individuals, those who received endocrine therapy alone demonstrated excellent five-year metastasis-free survival rates, reaching 94.7% (95% CI, 92.5 to 96.2). This finding underscores the potential to spare such patients from more aggressive treatments, irrespective of nodal status [20,42]. However, intriguing insights emerged from exploratory analyses within the MINDACT trial, particularly regarding age as a determining factor. For women younger than 50 years old, the addition of chemotherapy was associated with notable benefits. Their eight-year distant metastasis-free survival reached 93.6% (95% CI 89.3–96.3) with chemotherapy compared to 88.6% (83.5–92.3) without chemotherapy [42]. These findings underscore the nuanced application of MammaPrint and the importance of considering patient age in tailoring treatment decisions.

### 3.3. PAM50 Prosigna^®^

PAM50 has undergone diverse validation efforts, particularly on a prognostic level, through various studies, with a notable emphasis on its application within the ABCSG-8 trial (NCT00291759) and ATAC trial (ISRCTN 18233230) patient cohorts. These pivotal studies have collectively underscored the significance of PAM50 in effectively stratifying node-positive patients based on their risk of recurrence when treated with endocrine therapy, thus potentially sparing low-risk patients from unnecessary chemotherapy interventions [43,44,45,46]. At present, the predictive significance of Prosigna is being rigorously validated through the OPTIMA (Optimal Personalized Treatment of Early BC using Multi-Parameter Analysis) study (ISRCTN42400492) [47]. This international academic study is a partially blinded randomized trial designed with an adaptive approach. It specifically focuses on test-directed chemotherapy treatment and includes Luminal cases with pN0-2. In the OPTIMA trial, cases are randomized to either standard chemotherapy followed by endocrine therapy or undergo Prosigna genomic assay and be assigned to standard therapy, if high risk (scoring > 60), or endocrine therapy alone if low risk. This comprehensive validation process within the context of a carefully designed trial seeks to further establish the predictive power and clinical utility of Prosigna, particularly in aiding treatment decisions for Luminal breast cancer patients across various nodal statuses.

### 3.4. EndoPredict^®^

The prognostic utility of EndoPredict^®^ has undergone rigorous validation in clinical trials, most notably within the GEICAM 9906 trial, which focused on node-positive, chemotherapy-treated HR+/HER2− EBC cases. Additionally, the performance of EndoPredict^®^ has been assessed within the ABCSG 8 trial, specifically among postmenopausal patients with HR+ BC. Intriguingly, while it has demonstrated its ability to offer prognostic value, EndoPredict^®^ did not exhibit predictive capability concerning the response to different chemotherapy regimens [48,49]. Notably, a retrospective analysis compared the EPclin score between patients who underwent a combination of chemotherapy and endocrine therapy and those who exclusively received endocrine therapy. Interestingly, this analysis showed that the high-risk EPclin score, rather than the EP molecular score, was predictive of the potential benefits conferred by chemotherapy [50]. Furthermore, the utility of EndoPredict^®^ extends into a less explored domain, encompassing premenopausal patients. Among this group, a low-risk EPclin score was found to be associated with enhanced DRFS in patients who exclusively received adjuvant endocrine therapy [51].

### 3.5. Breast Cancer Index^®^

The clinical validation of BCI has emerged from a prospective–retrospective translational study of HR+ EBC patients enrolled in the IDEAL trial (BOOG 2006-05), which examined the predictive component of the BCI assay [52]. Specifically, the outcomes of randomized treatment with either an additional 5 years or 2.5 years of letrozole were significantly influenced by the classification based on BCI. Patients characterized by a high H/I ratio experienced substantial benefits from extended endocrine therapy lasting at least 5 years. This translated into a remarkable reduction of 58% and 66% in the relative risk of recurrence across the entire cohort and the subgroup treated with primary adjuvant aromatase inhibitors, respectively. However, patients with a low H/I ratio did not manifest a similar level of benefit [53]. These findings underscore the clinical utility of BCI not only in assessing the risk of recurrence but also in guiding the duration of endocrine therapy.

## 4. Current Guidelines on Gene Profiling Assays

The European Society for Medical Oncology (ESMO) and the ASCO guidelines presently refrain from favoring one specific genomic test over another. Instead, they underscore the collective value of these assays, particularly in intricate clinical scenarios where the selection of optimal adjuvant therapy remains uncertain, as exemplified in Luminal B with 1–3 positive axillary node disease [54]. ASCO guidelines [11] provide specific recommendations for the use of genomic assays based on patient characteristics and clinical circumstances. In postmenopausal women over the age of 50 with HR+/HER2− EBC, these guidelines suggest the consideration of Oncotype Dx, MammaPrint, or the Breast Cancer Index (BCI). This recommendation applies to patients who are either node-negative or have 1–3 node metastases. For premenopausal patients, the guidelines advocate the use of Oncotype Dx in node-negative HR+/HER2− cases. However, in the presence of 1–3 axillary lymph node metastases, chemotherapy is recommended irrespective of genomic assay results. Notably, the guidelines do not currently provide specific recommendations for the application of genomic tests in patients with four or more positive nodes [11]. The current ASCO and ESMO recommendations on gene profiling assays in early HR+/HER2− BC, together with the respective level of evidence and grade of recommendation, are summarized in Table 1 and Table 2, respectively. These guidelines reflect the evolving landscape of genomic testing in BC and aim to assist clinicians in making informed decisions, particularly in scenarios where traditional clinical parameters may not provide clear guidance.

## 5. Discussion of the Current Challenges and Opportunities

### 5.1. Outsourcing vs. ‘in-House’ Approaches

Regarding the implementation of multigene genomic assays, the distinction between an ‘in-house’ testing model and an outsourcing model has emerged as a pivotal consideration [55]. While both approaches share the overarching goal of providing clinically actionable insights, they differ fundamentally in their execution and implications [56]. The ‘in-house’ model, exemplified by assays like Prosigna/PAM50, is predicated on the idea of conducting gene expression profiling tests within the confines of a healthcare institution’s own laboratory facilities. This approach offers a measure of control over the testing process, allowing for direct oversight of procedures, quality control, and turnaround times (TAT). Furthermore, it affords the institution the flexibility to tailor testing protocols to its specific patient population and clinical objectives. In contrast, the outsourcing model, as epitomized by Oncotype Dx, requests the shipment of patient samples to external laboratories or service providers specializing in gene expression profiling. While this approach may offer the convenience of not requiring extensive in-house infrastructure or expertise, it comes with its own set of challenges. One of the primary considerations is the dependence on external providers, which can result in extended TAT and limited control over the testing process. This can be particularly pertinent in time-sensitive clinical scenarios where rapid decision-making is necessary [57].

### 5.2. Cost–Utility Analysis

The economic implications distinctly highlight the disparities between the models under consideration. Opting for ‘in-house’ testing, particularly when dealing with smaller patient volumes, can introduce financial burdens stemming from the need to acquire reagents and equipment that come with expiry dates [58]. This scenario demands the attainment of economies of scale to render the investment economically viable. Conversely, while outsourcing may introduce additional costs from external sources, it could offer a more financially prudent option for entities with lesser testing demands [59]. This approach circumvents the financial and logistical burdens tied to upholding an in-house testing framework. A pivotal inquiry in this context is determining the most fitting model that aligns with the clinical demands and limitations of each healthcare institution prior to the adoption of a genomic test. This decision is contingent upon various factors, including the volume of patients, proficiency in conducting the test and interpreting its outcomes, and the specific clinical settings where the test yields the most value [60]. The crux of this decision-making process involves weighing the benefits of ‘in-house’ control and adaptability against the ease and potential cost savings of outsourcing [61]. As EBC management advances, it is imperative for institutions to meticulously evaluate this choice to guarantee superior patient care.

### 5.3. Unmet Clinical Needs

One of the primary challenges lies in the integration of these assays into clinical decision-making, which hinges on the availability of clinical predictive information. Ongoing trials, like OPTIMA, hold promise in this regard, as they have the potential to refine the utility of gene expression profiling assays. This is especially crucial for subgroups that have traditionally posed challenges for existing genomic tests. For instance, premenopausal patients with 1–3 positive axillary lymph nodes have often presented a conundrum, as current assays, such as Oncotype Dx, may not offer conclusive guidance, and chemotherapy is the only advised approach. Exploring the potential of assays like Prosigna in such cases could prove useful [62]. Adding complexity to this landscape is the consideration of BC patients with four or more positive nodes, representing another intriguing subgroup for study. Furthermore, there is an emerging interest in the application of gene expression profiling assays in the neoadjuvant/preoperative setting, particularly among Luminal BC patients [63,64]. This domain warrants further investigation, as it has the potential to optimize therapeutic choices and individualize treatment approaches. Moreover, gene profiling assays have mainly focused on early BC, while they might provide interesting information regarding high-risk and high-grade patients diagnosed with locally advanced, relapsed, and metastatic BC [65]. As gene expression profiling assays progress in clinical application, practical considerations arise, notably in deciding whether to rely solely on one test, such as Oncotype Dx or Prosigna, or explore a combination of multiple assays. This decision poses crucial questions regarding clinical effectiveness and the practical challenges associated with implementation and reimbursement. While it is crucial to continue pursuing advancements in genomic testing, addressing these practicalities is essential to ensure that the benefits of such testing are accessible to a broader patient population.

## 6. Conclusions

In conclusion, the evolving landscape of gene expression profiling assays in EBC underscores the need to bridge the gap between technological capability and clinical relevance. Despite some striking differences, currently available tests have several common advantages and disadvantages. The prospective validation of Oncotype Dx for both pre- and postmenopausal patients, along with the availability of the RSClin tool that integrates various clinicopathological features, yields robust results. However, cost constraints and the need for sample externalization can impede its widespread adoption. MammaPrint faces similar limitations, despite being a potent FDA-approved and CE-IVD-marked assay, using data from patients with a 20-year follow-up. Notably, MammaPrint offers versatility by accommodating both frozen and FFPE samples. PAM50 Prosigna is renowned for its intrinsic subtyping and risk stratification while offering the possibility of being processed in-house. Nevertheless, it necessitates further validation to ascertain its predictive capability regarding response to diverse chemotherapy regimens. Conversely, EndoPredict^®^ exhibits robust prognostic validity, yet additional validation is necessary to determine its efficacy in predicting chemotherapy response. Lastly, BCI demonstrates remarkable clinical utility in both recurrence risk assessment and guiding endocrine therapy duration. However, the centralization of the test may lead to increased TAT. Addressing these specific strengths and limitations of each assay is crucial for informed decision-making in clinical practice. This involves delineating the precise clinical scenarios where these assays offer the most significant advantages, expanding their applications to previously challenging patient populations, and addressing the practicalities that can facilitate their seamless integration into clinical practice. By doing so, we can harness the full potential of these assays to enhance the precision and efficacy of BC diagnosis and treatment.

## Figures and Tables

**Table 1:** Current ASCO recommendations on gene profile assays in HR+/HER2- early breast cancer. Adapted from Andre et al. [11]. BCI, Breast Cancer Index; evidence quality: high (++), intermediate (+); strength of recommendation: strong (++), moderate (+); insufficient evidence to recommend a biomarker (−).

Lymph Node Status	Premenopausal or Age ≤ 50 Years (Evidence Quality/Strength of Recommendation)	Postmenopausal or Age > 50 Years (Evidence Quality/Strength of Recommendation)
Node-negative	Oncotype Dx (++/++)	Oncotype Dx (++/++) MammaPrint (+/++) EndoPredict^®^ (+/+) Prosigna (+/+) BCI (+/+)
1–3 positive nodes	(−)	Oncotype Dx (++/++) MammaPrint (+/++) EndoPredict^®^ (+/+) BCI (+/+)
≥4 positive nodes	Insufficient evidence to recommend a biomarker	

**Table 2:** Current ESMO recommendations on gene profiling assays in HR+/HER2- early breast cancer. Adapted from Cardoso et al. [54]. Level I of evidence means that the evidence derives from at least one large randomized controlled trial of good methodological quality (low potential for bias) or meta-analyses of well-conducted randomized trials without heterogeneity. Grade of recommendation A equals strong evidence for efficacy with a substantial clinical benefit, while grade of recommendation B means there is strong or moderate evidence for efficacy but with a limited clinical benefit (general recommendation).

	Level of Evidence	Grade of Recommendation
**First-generation (Oncotype Dx and MammaPrint)** Prognostic and predictive value (Neo)adjuvant chemotherapy is indicated if high risk or high score (predictive value) Can be carried out in biopsy or surgical specimen	**I**	**A**
**Second generation (Prosigna, EndoPredict** **^®^** **)** Prognostic value (Neo)adjuvant chemotherapy is indicated if high risk or high score Can be carried out in biopsy or surgical specimen	**I**	**B**
**First-generation (Breast Cancer Index)** Prognostic and predictive value Yes/no prediction of benefit from extended (>5 years) endocrine therapy Can be carried out in biopsy or surgical specimen	**I**	**B**

## Data Availability

No new data were created in this study. Data sharing is not applicable to this article.
